# Emerging Role of PARP Inhibitors in Metastatic Triple Negative Breast Cancer. Current Scenario and Future Perspectives

**DOI:** 10.3389/fonc.2021.769280

**Published:** 2021-11-25

**Authors:** Giacomo Barchiesi, Michela Roberto, Monica Verrico, Patrizia Vici, Silverio Tomao, Federica Tomao

**Affiliations:** ^1^ Dipartimento di Scienze Radiologiche, Oncologiche ed Anatomo Patologiche, Università di Roma Sapienza, Rome, Italy; ^2^ UOSD Sperimentazioni Di Fase IV, Istituto di Ricerca e Cura a Carattere Scientifico (IRCCS) Regina Elena National Cancer Institute, Rome, Italy; ^3^ Gynecologic Oncology Program, European Institute of Oncology, Istituto di Ricerca e Cura a Carattere Scientifico (IRCCS), Milan, Italy; ^4^ Maternal and Child Department, Sapienza University of Rome, Rome, Italy

**Keywords:** triple negative, metastatic breast cancer, PARP inhibitors, olaparib, talazoparib, BRCA1/2

## Abstract

Triple negative tumors represent 15% of breast cancer and are characterized by the lack of estrogen receptors, progesterone receptor, and HER2 amplification or overexpression. Approximately 25% of patients diagnosed with triple negative breast cancer carry a germline BRCA1 or BRCA2 mutation. They have an aggressive biology, and chemotherapy has been the mainstay of treatment for a long time. Despite intensive therapies, prognosis is still poor, and many patients will eventually relapse or die due to cancer. Therefore, novel targeted agents that can increase the treatment options for this disease are urgently needed. Recently, a new class of molecules has emerged as a standard of care for patients with triple negative breast cancer and germline BRCA1 or BRCA2 mutation: poly (ADP-ribose) (PARP) inhibitors. In the first part of the review, we summarize and discuss evidence supporting the use of PARP inhibitors. Currently, two PARP inhibitors have been approved for triple negative metastatic breast cancer—olaparib and talazoparib—based on two phase III trials, which showed a progression-free survival benefit when compared to chemotherapy. Safety profile was manageable with supportive therapies and dose reductions/interruptions. In addition, other PARP inhibitors are currently under investigation, such as talazoparib, rucaparib, and veliparib. Subsequently, we will discuss the potential role of PARP inhibitors in the future. Clinical research areas are investigating PARP inhibitors in combination with other agents and are including patients without germline BRCA mutations: ongoing phase II/III studies are combining PARP inhibitors with immunotherapy, while phases I and II trials are combining PARP inhibitors with other targeted agents such as ATM and PIK3CA inhibitors. Moreover, several clinical trials are enrolling patients with somatic BRCA mutation or patients carrying mutations in genes, other than BRCA1/2, involved in the homologous recombination repair pathway (*e*.*g*., CHECK2, PALB2, RAD51, *etc.*).

## Introduction

Although survival rates are constantly improving because of the current strategies of primary/secondary prevention and the availability of innovative and personalized therapeutic challenges, breast cancer (BC) is still the most frequent malignant neoplasia and the leading cause of cancer-related lethality among women worldwide today. Moreover, it is also the second most common cancer in the world (1–3). According to these data, BC constitutes one of the greatest health emergencies in Western countries today, pushing the health authorities to commit enormous resources to fight against this cancer. In 2020, an estimated 276,480 new cases of female breast cancer will be diagnosed in the US, and 42,170 metastatic BC patients are expected to die due to this disease. Some biological, epidemiological, and clinical aspects of BC deserve to be better investigated in order to explain the many differences occurring in clinical practice: geographic distribution of BC, reasons for the increasing early onset in young women, unexpected severe poor outcome in some patients with favorable prognostic factors, different levels of availability of targeted agents, and frequent occurrence of orphan drug diseases. In this context, a better understanding of the molecular portraits of BC in the last years has played a prominent role in order to improve our knowledge about a tailored BC clinical approach. Moreover, this speculative and investigative strategy could identify other novel molecular targets (beyond estrogen receptors, HER-2, and PIK3CA) that could better inhibit BC growth and diffusion, mainly in association with currently used drugs. BC is a heterogeneous disease, with different profiles of gene expression and amplifications determining great differences in prognosis and therapeutic strategies (4–6). In 2000, Perou et al. showed, by analyzing 8,102 different genes, that the phenotypic diversity of BC corresponded to specific gene expression profiles (4). This study identified four different molecular portraits that might be related to the specific molecular features of mammary epithelial biology: ER+/luminal-like, basal-like, Erb-B2 enriched, and normal breast.

Triple negative breast cancer (TNBC) represents about 14–16% of all BC patients and is characterized by lack of estrogen receptor (ER), progesterone receptor (PR), and HER2 expression. Lehmann et al. analyzed the gene expression profiles of BC patients by analyzing 21 databases and suggesting that a high and unexpected heterogeneity distinguishes TNBC from other BC tumors (7, 8). The authors suggested six different TNBC subtypes: two basal-like types (BL 1–2), a mesenchymal type (MES), an immunomodulatory, a mesenchymal stem-like, and a luminal androgen receptor (LAR). According to these gene analyses, TNBC shows partial correlations with basal-like type BC (discordance of about 20–30%). In a different study, other authors proposed four distinguished TNBC subtypes: LAR, MES, basal-like immunosuppressed, and basal-like immune-activated (9). However, despite the increasing knowledge of TNBC biology, there are no evidence to support their use in clinical practice for treatment selection. TNBC is an aggressive disease and frequently associated with early and distant recurrence, occurrence of visceral metastases, and higher risk of death compared to other BC types. Moreover, metastatic recurrence is constantly related with a short progressive disease and premature occurrence of death (usually, the median survival of advanced TNBC is not longer than 12 months) (10–14). TNBCs are usually basal-like, and they express a claudin-low condition and present high levels of cancer stem cells (15–18), which could explain their aggressive clinical behavior.

A chemotherapeutic approach has been considered for a long time as the most active and efficient systemic treatment for metastatic TNBC (19–24). Because TNBC frequently demonstrates an important immunogenic profile, a high number of tumor-infiltrating lymphocytes, and a high level of PD-L1 expression (25, 26), it has been possibly considered the most suitable BC subtype for immunotherapy. In fact, the combination of chemotherapy and immune-checkpoint inhibitors (ICIs) has shown superior efficacy in terms of progression-free survival (PFS) and overall survival (OS) when compared to chemotherapy in monotherapy and currently represents the standard of care for patients with PD-L1 positive metastatic TNBC (17, 27). In addition, TNBC could benefit also from other chemotherapeutic drugs, such as capecitabine and eribulin, in different settings (28–31).

The possibility to treat TNBC with other novel targeted agents have recently emerged in order to evaluate the relationship between this BC subtype and the occurrence of deleterious *BRCA1* and *BRCA2* mutations. On one hand, approximately 70% of BRCA1-2-mutated BC patients express TNBC subtype, and on the other hand, 10–20% of all TNBC are BRCA1/2 mutation carriers (32–34), regardless of family history (35).

According to these data and considering that some PARP inhibitors (PARPib) are FDA-approved (olaparib and talazoparib) for the treatment of *BRCA*-associated BC (36–39), an increased interest has emerged to evaluate their activity and safety specifically in TNBC patients with BRCA1/2 mutations. Moreover, other PARPib (niraparib, rucaparib, and veliparib) are being investigated in large randomized clinical trials in order to assess their activity as single agents or in combination with other drugs (chemotherapy, ICIs, and targeted molecules).

This review summarizes the current evidence supporting the use of PARPib in BRCA-mutated TNBC patients and focuses on new potential strategies to improve their outcomes and therapeutic opportunities.

## The Rational Behind PARP Inhibitors: The Synthetic Lethality

DNA damage represents one of the leading processes of carcinogenesis and can occur through different mechanisms: single-strand breaks (SSB), helix-distorting damage, replication errors, and double-strand breaks (DSB). Specifically, DSB are considered one of the most cytotoxic types of DNA damage, so it is not a surprise that normal cells have developed multiple pathways to repair it. Among the DSB repair pathways, a key role is played by homologous recombination (HR) and nonhomologous end-joining (NHEJ) (40, 41). On the other hand, SSB, helix distorting damage, and replication errors are corrected by base excision repair, nucleotide excision repair, and mismatch repair, respectively.

**Figure 1:**
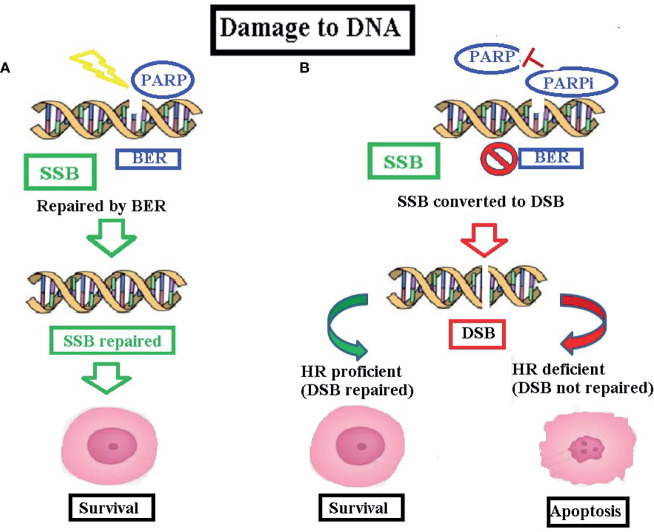
**(A)** PARP mechanism of action: PARP enzymes are key components in base excision repair, a DDR pathway which deals with SSB. In case of SSB DNA damage, PARP enzymes attach to the damaged DNA and allow NAD+ to bind to its active site. ADP-ribose moieties from NAD+ are transferred to target proteins (PARylation), which recruit single-strand DNA repair effectors. After the DNA damage has been repaired, PARP autoPARylates, returning to a catalytic state of inactivation. **(B)** PARP inhibitor mechanism of action: the synthetic lethality—PARPib are a class of molecules which prevent SSB repair. If SSB damage cannot be repaired, the immediate consequence is DSB formation. In cells with a proficient HRR pathway, HRR effectors (among which BRCA1 and BRCA2 play a crucial role) repair DSB, allowing cell survival. In tumor cells with HRR deficiency treated with PARPib, concomitant inhibition of base excision repair and HRR lack of function cause a progressive accumulation of DNA alterations which ultimately leads to cell apoptosis. DDR, damaged DNA repair; DSB, double-strand breaks; HRR, homologous recombination repair; PARPib, PARP inhibitors; SSB, single-strand breaks.

Poly(ADP-ribose) polymerases (PARP) are a large family of multifunctional enzymes with a key role in base excision repair mechanism (42). Eighteen members have been identified, among which PARP-1 is the most important, while PARP2 and PARP3 are less involved. PARP-1 is essential for SSB repair, and it plays a dominant role in genome integrity (43). In particular, PARP-1 detects the damage of DNA and catalyzes the so-called PARylation, which is the addition of a poly-ADP-ribose (PAR) chain to target proteins in order to recruit additional repair factors on the damaged DNA (**Figure 1A**) (44, 45).

More recently, increasing evidences have shown that PARP can also be involved in DSB repair: PARP-1 recruits MRE11 and NS1 enzymes which are crucial in HR pathways (46) by opening the chromatin structure to give access to repair proteins.

Cancer cells affected by deleterious mutation in breast cancer susceptibility genes 1 or 2 (*BRCA* 1/2) are deficient in the DNA DSB repair. In fact, both BRCA1 and BRCA 2 are key components in the homologous recombination repair (HRR) pathway (47). BRCA 1 is a multifunctional enzyme with a direct involvement in HRR: with CHK2, it is initially responsible for signal transduction; after that, DNA double strand damage is recognized by ATM and ATR (47). Subsequently, it acts by forming a structure which organizes repair proteins at the DNA repair site (48, 49). BRCA 2, on the contrary, recruits RAD51 (a recombinase) at the DNA repair site (50). Therefore, tumors with BRCA 1 and BRCA2 inactivation are highly dependent on the repair pathway for SSB (51–53). Consequently, if other events occur that can impair DNA damage repair, the damage can lead to a progressive accumulation of DNA alterations which can ultimately lead to apoptosis (**Figure 1B**) (54, 55).

The aforementioned mechanism represents the core concept of synthetic lethality: an interaction between two genes in which the mutation of either gene alone is compatible with viability, while the simultaneous mutation of both genes causes death (56–58). PARPib are the first clinically approved drugs designed to exploit synthetic lethality, showing promising activity in patients with *BRCA* deficient tumors (53).

PARPib exert their functions through different systems: initially, it was believed that their principal mechanism of action consisted of “catalytic inhibition”: a competing bind to the PARP1 and PARP2 catalytic domains which displaces nicotinamide adenine ribonucleoside (NAD+) from its active site, thus preventing the recruitment of single-strand DNA repair effectors (59, 60). More recently, it has been demonstrated that PARPib act mostly by inhibiting the PARylation mechanism which induces the trapping at the site of DNA damage, the activation of effector genes, and consequently the interruption of the replication fork by leading to a DSB damage responsible for a cytotoxic effect (61). Accordingly, preclinical models showed that trapping DNA on PARP could be more effective in inducing cell death than catalytic enzyme alone (43, 60). Thus, in tumors harboring a defect in the HRR pathway, contemporary inhibition of PARP enzymes causes the accumulation of unpaired damages, leading to tumor cell death. On the contrary, healthy cells can be spared, thus providing a clinical benefit in patients with *BRCA* 1 or 2 mutation (62). The capacity of PARP trapping is different among PARPib and is independent from catalytic inhibition (43, 60, 63, 64). This difference can partially explain the different clinical activity and safety profile of PARPib.

At this time, two PARPib have been approved for the treatment of patients with TNBC in the metastatic setting: olaparib and talazoparib. Olaparib is a small molecule, which was initially described as a PARP-1 and PARP-2 inhibitor but for which recent data showed also a potent PARP-3 inhibition (65). Talazoparib, on the contrary, is a potent PARP inhibitor, with both strong catalytic inhibition and PARP trapping potential (preclinical models showed that the trapping potential of talazoparib is 100 times higher than the other PARPib) (63).

## Clinical Evidence of PARP Inhibitors in Triple Negative Breast Cancer

Olaparib and talazoparib are currently approved as monotherapy for the treatment of metastatic TNBC harboring a germline *BRCA* (g*BRCA*) 1 or 2 mutation based on the results of two phase III trials: OlympiAD (37, 66) and EMBRACA (36).

### OlympiAD Trial

The OlympiAD trial enrolled 302 metastatic breast cancer patients with both triple negative (TN) (49.8%) and hormone receptor positive (HR+) HER2 negative (50.2%) tumors. Not more than two lines of chemotherapy for metastatic disease were permitted. Pre-treatment with platinum was allowed, but the last dose should have been administered at least 12 months before randomization. The patients were randomized in a 2:1 ratio to receive olaparib, 300 mg bid, monotherapy or treatment physician’s choice (TPC) among capecitabine, eribulin, or vinorelbine. The primary endpoint was PFS. The secondary endpoint included OS, overall response rate (ORR), and health-related quality of life (HRQoL). The primary analysis showed that PFS was significantly longer in the olaparib arm than the standard chemotherapy (7.0 *vs*. 4.2 months; HR: 0.58; 95% CI: 0.43 to 0.80; *p* < 0.001). ORR was also higher in the olaparib group than in the standard chemotherapy group (59.8 *vs*. 29.8%). OS, on the contrary, did not differ from the two arms (HR for death, 0.90; 95% CI, 0.63 to 1.29; *P* = 0.57), but the trial was not powered to assess OS differences. In the forest plot, HR was lower in the TN subgroup than in the HR+ subgroup (0.43 *vs*. 0.82). Finally, olaparib had a good safety profile: there were fewer grade 3 events and fewer discontinuations related to an adverse event in the olaparib arm than in the chemotherapy arm. The side effects reported were comparable to previously published phase I and II trials with anemia, nausea, vomiting, fatigue, headache, and cough occurring more frequently in the olaparib group than in the standard therapy group (37).

The planned study final analysis with OS update has been recently published (66). Overall, OS was not improved by olaparib treatment compared to standard chemotherapy (19.3 months with olaparib *versus* 17.1 months with TPC HR: 0.90, 95% CI: 0.66–1.23; *p* = 0.513); however, when patients were stratified according to pre-defined subgroups, an OS benefit was observed in patients who had not received prior chemotherapy for metastatic disease (first-line treatment, 22.6 *versus* 14.7 months; HR: 0.51; 95% CI: 0.29–0.90). Safety data were also updated: no new findings were reported. Overall, the incidence of grade 3 adverse events was 38%, while 5% of patients discontinued olaparib because of toxicity.

Another important secondary endpoint of the OlympiAD trial was the quality of life (QoL) of the patients. Investigators employed the European Organisation for Research and Treatment of Cancer Quality-of-Life Questionnaire Core 30-item module (EORTC QLQ-C30) to assess patient global health status/QoL. The final results (67) showed a significant QoL improvement in the olaparib arm compared to the TPC arm with a mean change of 3.9 (standard deviation 1.2) *versus* -3.6 (2.2), a difference of 7.5 points (95% confidence interval, CI: 2.48, 12.44; *p =* 0.0035). In addition, for EORTC QLQ-C30 symptoms and functioning subscales, only the nausea/vomiting symptom score was worse in the olaparib arm than in the TPC arm (across all visits compared with baseline) (68).

An extended follow-up exploratory analysis of the OlympiAD trial was presented at the San Antonio Breast Cancer Symposium in December 2019 (69). The median follow-up was 18.9 *vs*. 15.5 months in the olaparib *vs*. the TPC arms, respectively. Median study treatment duration was 8.3 months in the olaparib arm *vs*. 3.5 months in the TPC arm, and in the olaparib arm, 8.8% of patients received the treatment for more than 3 years, while no one did in the TPC arm. The results of the extended follow-up confirmed previously published results: no OS differences were registered between the two arms in the overall population (19.3 months for olaparib *vs*. 17.1 months in the TPC arm, HR: 0.84, 95% CI: 0.63–1.12), but an OS benefit was detected in the subgroup of patients treated with olaparib who had not received chemotherapy for metastatic setting (first-line treatment: OS 22.6 month in the olaparib arm *vs*. 14.7 months in the TPC arm, HR: 0.54, 95% CI: 0.32–0.92). In first-line subgroup, 40.8% of patients in the olaparib arm were alive at 3 years compared with 12.8% of patients in the TPC arm. No new safety data and no cumulative toxicity occurred at the extended follow-up analysis, confirming good olaparib tolerability even in long-term exposure.

### EMBRACA Trial

The phase III EMBRACA trial was an open-label, randomized trial, comparing talazoparib *versus* choice of standard chemotherapy of the physician (capecitabine, eribulin, gemcitabine, or vinorelbine) in pretreated locally advanced (not amenable to curative treatment) or metastatic *BRCA*1/2 mutated breast cancer (36). A total of 431 patients were randomized in a 2:1 ratio to receive talazoparib at a dose of 1.0 mg daily (*n* = 287) *vs*. standard chemotherapy (*n* = 144). Forty percent of the enrolled patients were TN. No more than three previous chemotherapy regimens were admitted. Patients must have had previously received anthracyclines and taxanes, unless clinically contraindicated. Previous platinum-based adjuvant or neoadjuvant chemotherapy was admitted only if the patients had a disease-free interval of at least 6 months from the last platinum dose. The primary endpoint was PFS by blinded independent central review. The secondary endpoints were OS and ORR. Safety and patient-reported outcomes were also assessed.

At a median follow-up of 11.2 months, the EMBRACA trial met its primary endpoint: the median PFS was significantly higher in the talazoparib arm (8.6 months; 95% CI, 7.2–9.3) than in the standard chemotherapy arm (5.6 months; CI, 4.2–6.7). The HR was 0.54 (95% CI, 0.41–0.71; *p* = 0.001), and it was confirmed by an independent radiologic review. The PFS HRs were consistent among subgroups, specifically, for HR+ and TN. The PFS HR was 0.47, 95% CI: = 0.32 to 0.71 for HR+/HER2− and 0.60, 95% CI: = 0.41 to 0.87 for TN (32, 33). The response rate by the investigators was 62.6% in the talazoparib arm compared with 27.2% in the chemotherapy arm.

At interim analysis, the median OS was longer in the talazoparib arm (22.3 months) than in the chemotherapy arm (19.5 months), but it did not reach statistical significance (HR: 0.76; 95% CI, 0.55–1.06, *p* = 0.11). Data about safety showed that the most common all-grade adverse events for talazoparib were anemia, fatigue, and nausea, while for chemotherapy nausea, fatigue, and neutropenia were more frequent. Grade 3 or 4 hematologic adverse events occurred in 55 *vs*. 36.1% of patients in the talazoparib and standard chemotherapy arms, respectively. Grade 3 non-hematologic adverse events occurred in 32 *vs*. 38% of patients in the talazoparib arm and in the standard chemotherapy arm, respectively. However, discontinuation rate due to an adverse event was low: 5.9% in the talazoparib group *vs*. 8.7% in the standard chemotherapy group. Moreover, talazoparib significantly delayed the onset of a clinically meaningful deterioration of global health status of QoL questionnaire, and it also significantly delayed deterioration according to breast symptom scale compared to chemotherapy (70).

In the final OS analysis, published after that 75% of the events occurred (324 patients), talazoparib showed no OS benefit compared to chemotherapy: median OS was 19.3 months (16.6–22.5 months) *versus* 19.5 months (17.4–22.4 months); HR: 0.848 (95% CI: 0.670–1.073; *P* = 0.17). A possible explanation for the lack of OS benefit relies on subsequent treatment that could have impaired the analysis: 32.6% of patients randomized to TPC received a PARP inhibitor in later lines of treatment (at the time of EMBRACA publication, olaparib had already been approved for metastatic breast cancer patients harboring g*BRCA* 1/2 mutation) (71).

More recently, a Cochrane metanalysis investigated the efficacy of PARPib in metastatic breast cancer patients with *BRCA* 1 or 2 mutations (72). The primary outcome was OS, while the secondary outcomes were PFS, tumor response rate, and safety. The authors included five trials involving 1,474 patients. PARPib showed a small OS benefit: HR: 0.87 (95% CI: 0.76 to 1.00; *P* = 0.05; high-certainty evidence), with no significant heterogeneity (*I* ^2^ = 0%, *P* =0.81). Unfortunately, subgroup analysis could not be performed because data were not available for the included trials. On the contrary, PARPib significantly prolonged PFS with a HR of 0.63 (95% CI: 0.56 to 0.71; *P* < 0.00001; high-certainty evidence), with no significant heterogeneity (*I* ^2^ = 2%, *P* = 0.39). For patients with TNBC (*N* = 664, four randomized controlled trials, RCTs), there was evidence of PFS benefit on pooling of studies (HR: 0.61, 95% CI: 0.47 to 0.80; *P* = 0.0003), with moderate heterogeneity (*I* ^2^ = 44%, *P* = 0.15).

In addition to olaparib and talazoparib, other PARPib are currently under investigation in TNBC: rucaparib, niraparib, and veliparib. Of note is that the phase III Bravo trial, which investigated the role of niraparib *versus* TPC in *BRCA* mutated breast cancer, was prematurely closed because of high discontinuation rate in the control arm (the patients enrolled in the control arm did not continue the trial long enough to receive their first radiological scan, which is required to assess disease progression, resulting in an unusually high rate of censoring) (68). A complete list of other published trials (73) and monotherapy ongoing trials of PARPib in TNBC is summarized in **Tables 1**, **2** (74, 75, 80, 81).

**Table 1:** Published trial with PARPib monotherapy.

Trial	*N*	Patients	Triple negative patients	Arms	Endpoints	Results
OlympiAD (39, 68, 72, 73)	302 (phase III)	gBRCA mutated, pretreated (≤2 lines of chemotherapy) HER2 neg mBC	49.8%	Olaparib 300 mg bid *versus* TPC (R 2:1)	Primary endpoint: PFS Secondary endpoints: ORR; OS, safety; HrQoL	Primary endpoint: PFS = 7.0 *vs*. 4.2 m; HR: 0.58; 95% CI: 0.43 to 0.80; *p* < 0.001 Secondary endpoints: ORR = 59.8 *vs*. 29.8% OS (final) = 19.3 *vs*. 17.1 m HR: 0.90, 95% CI: 0.66–1.23; *p* = 0.513 Safety: lower grade 3 events rate with olaparib than TPC (38 *vs*. 49%) HRQoL significantly improved with olaparib
EMBRACA (38, 74)	431 (phase III)	gBRCA mutated, pretreated (≤3 lines of chemotherapy) HER2 neg mBC	40%	Talazoparib 1 mg *vs*. TPC	Primary endpoint: PFS Secondary endpoints: ORR; OS, safety; HrQoL	Primary endpoint: PFS = 8.6 *vs*. 5.6 m, HR: 0.54, 95% CI: 0.41–0.71; *p* = 0.001 Secondary endpoints: ORR: 62.6 *vs*. 27.2% OS (final) = 19.3 *vs*. 19.5 m HR: 0.85, 95% CI: 0.670–1.073; *P* = 0.17 Safety = higher grade 3 hematological events with talazoparib (55 *vs*. 36.1%); lower grade 3 non-hematological events with talazoparib (32 *vs*. 38%) HRQoL significantly improved with talazoparib
ABRAZO (75)	84 (phase II)	Pretreated gBRCA mBC with CR or PR after platinum chemotherapy (cohort 1) or platinum-naïve patients who had received ≤3 cytotoxic chemotherapies (cohort 2)	59% cohort 1; 17% cohort 2	Talazoparib 1 mg *vs*. placebo	Primary endpoint: ORR Secondary endpoints: CBR, PFS, DoR	Primary endpoint: ORR 28% (21% cohort 1, 37% cohort 2); 2 CRs, 21 PRs, 36 SD Median DoCR: 4.9 months (5.8 months cohort 1, 3.8 months cohort 2) CBR: 35% (27% cohort 1, 46% cohort 2) ORR: 26% (TNBC), 29% (HR+), Median PFS: 4.0 months (cohort 1) and 5.6 months (cohort 2) Median OS: 12.7 months (cohort 1) and 14.7 months (cohort 2) Grade ≥ 3 hematologic: 58% (cohort 1) and 60% (cohort 2); grade ≥ 3 Non-hematologic 27% (cohort 1) and 31% (cohort 2) Cohort 1: association between higher ORR and longer median PFS with longer platinum-free interval

BC, breast cancer; CBR, clinical benefit rate; DoR, duration of response; gBRCA, germline BRCA; HRQoL, health-related quality of life; mBC, metastatic breast cancer; ORR, overall response rate; OS, overall survival; PFS, progression-free survival; TN, triple negative; TPC, treatment physician’s choice.

**Table 2:** Ongoing clinical trial with PARPib monotherapy.

Trial	PARP inhibitor	Setting	Trial characteristics	End points	Study start date (study end)
ABC NCT02826512 (76)	Niraparib monotherapy (300 mg QD continuously)	LA incurable or metastatic Her2 negative, BRCA-1 like BC	Phase II, single-arm niraparib, ≤1 prior line of therapy for advanced BC N. patients: 39	Primary: PFS Secondary: ORR, duration of response, toxicity	Status: recruitment ongoing Start: May 2018 End: Aug 2022
BRAVO NCT01905592 (77)	Niraparib 300 mg once daily continuously *vs*. TPC (vinorelbine or eribuline or capecitabine)	Previously treated, Her2-negative, gBRCA mutated, metastatic BC, ≤2 previous therapies for metastatic disease	Phase III, randomized, open-label, multicenter, controlled N. patients: 215	Primary: PFS Secondary: OS, health-related QoL	Status: active, not recruiting Start: June 2013 End: Oct 2019 Study prematurely closed because of highly censored patients in the control arm
LUCY NCT03286842 (78)	Olaparib monotherapy 150 mg twice daily continuously	Metastatic Her2-negative, gBRCA or sBRCA mutation, ≤2 previous therapies for metastatic disease	Phase IIIb, open-label, multicenter N. patients: 256	Primary: PFS in real-word setting in gBRCA 1/2 mutated Secondary: OS in gBRCA mutated, TFST in gBRCA mutated, TSST in gBRCA mutated; TDT in gBRCA mutated; PFS2 in gBRCA mutated; CRR in gBRCA mutated; DoCR in gBRCA mutated, safety and tolerability	Status: Start: Jan 2018 End: Nov 2020
NCT02401347 (79)	Talazoparib 1 mg/day	Pretreated metastatic TN with HRD based on Miriad HRD assay	Phase II not randomized; *N* = 40	Primary endpoint: ORR; secondary: CBR, PFS, safety	Status: active Start: August 2015 End: December 2022 Recruiting

BC, breast cancer; CBR, clinical benefit rate; DoR, duration of response; gBRCA, germline BRCA; HRD, homologous recombinant deficiency; HRQoL, health-related quality of life; ORR, overall response rate; OS, overall survival; PFS, progression-free survival; TN, triple negative; TPC, treatment physician’s choice; TFST, time to first subsequent treatment or death; TSST, time to second subsequent treatment or death.

### Future Perspective and Ongoing Clinical Trials

Despite the fact that the role of PARPib as a therapeutic milestone is now confirmed in the management of BRCA-mutant TNBC, approximately 50% of patients progressed during treatment (76). From preclinical studies, four principal mechanisms of resistance have been identified (77): (i) the influence of cellular availability of the inhibitor, mainly by overexpression of drug-efflux transporter genes; (ii) direct impact on the activity and abundance of PAR chains due to PARP1 mutations that diminish trapping of the protein on DNA or the loss of PAR glycohydrolase, which is responsible for the degradation of PAR chains; (iii) the occurrence of “reversion mutations” that lead to the reactivation of both BRCA1/2 function and of HR by the activation of a specific protein complex (53BP1–RIF1–Shieldin axis); and (iv) influence of replication fork protection, mainly due to the attack by MRE11 and MUS81 nucleases.

Although the clinical relevance of this issue needs to be proven, some new drugs are engineered to target the acquired vulnerabilities of resistant tumors, thus restoring PARPib sensitivity.

Overall, PARPib showed improved PFS and response rate compared with standard chemotherapy, but no difference in OS was observed in those studies (72). Thus, the development of new agents and/or combination strategies are urgently needed to overcome PARPib resistance and to better understand TNBC molecular aspects. Several ongoing clinical trials aiming at evaluating the safety and efficacy of PARPib in combination with immune checkpoint inhibitors (78, 79, 82–90), chemotherapy (91–94), or target agents (95–100) for advanced BC (including TNBC) are summarized in **Tables 3**–**5**. Particularly promising are the data that emerged with combinations of PARPib and immunotherapy according to the durable response rates (101–103).

**Table 3:** Clinical trial with PARPib plus immunotherapy.

Trial	PARP inhibitor	Setting	Trial characteristics	End points	Study start date (study end)
TOPACIO NCT02657889 (83)	Niraparib up to 300 mg PO dd 1-21 + Pembrolizumab 200 mg i.v. every 21 days	Advanced or metastatic triple negative breast cancer or recurrent ovarian cancer	Phase I (niraparib dose escalation)/phase II study N. patients: 122	Primary: phase I: - niraparib DLTs, toxicity - ObRR Secondary: phase I: - safety and tolerability, DOR, PFS, OS, PK	Status: active, not recruiting Start: Mar 2016 End: Mar 2020
MEDIOLA NCT02734004 (84)	Olaparib 300 mg b.i.d. + MEDI4736 (durvalumab) 1,500 mg i.v. every 28 days from 5 weeks *vs*. olaparib 300 mg b.i.d. + MEDI4736 (durvalumab) 1,500 mg i.v. every 28 days *vs*. olaparib 300 mg b.i.d. + MEDI4736 (durvalumab) 1,500 mg i.v. every 28 days and beacizumab every 14 days	Advanced solid tumors (NSCLC, gBRCAm TNBC, gBRCAm ovarian cancer, gastric cancer)	Phase I/II, multicenter, N. patients: 264	Primary: DCR, ORR, safety, Secondary: PFS, OS, DoR, pharmacokinetic	Status: active, not recruiting Start: Apr 2016 End: Apr 2021
DORA NCT03167619 (85)	Olaparib 300 mg b.i.d. monotherapy *vs*. olaparib same doses + durvalumab i.v. every 28 days	Inoperable, LA or metastastic TN adenocarcinoma, previously treated with first- or second-line platinum-based therapy, with clinical benefit	Phase II, randomized, multicenter study N. patients: 60	Primary: PFS Secondary: OS, safety and tolerability, ORR,	Status: active, recruiting Start: Oct 2018 End: Dec 2020
NCT02484404 (86)	Olaparib + cediranib + MEDI4736 (durvalumab)	Advanced solid tumors (ovarian, TN, lung, prostate, CRC)	Phase I-II; *N* = 384	Primary: safety, tolerability, ORR; Secondary; PFS	Status: active Start: Jun 2015 End: Dec 2022
DOLAF NCT04053322 (87)	Olaparib 300 mg b.i.d. + durvalumab 1,500 mg i.v. every 28 days from cycle 2 + fulvestrant 500 mg i.m. cycle 1 days 1 and 15, from cycle 2 day 1 every 28 days	HR-positive, Her2-negative, LA or metastatic breast cancer with BRCA gene alterations or with HRR gene alterations or with MSI status	International, multicenter, phase II, single arm study N. patients: 158	Primary: PFSR Secondary: Safety, OS, ORR, DoR, PFS	Status: active, recruiting Start: Aug 2019 End: Aug 2025
Olaparib and atezolizumab NCT02849496 (88)	Olaparib b.i.d. dd 1–21 every 21 days monotherapy (arm I) or olaparib + atezolizumab every 21 days (arm II)	LA or metastatic, HDR deficient, Her2-negative BC	Phase II open-label, randomized N. patients: 72	Primary: PFS Secondary: ORR, DoR,	Status: active recruiting Start: Nov 2016 End: Aug 2020
NCT04683679 (89)	Pembrolizumab + RT +7- olaparib 300 mg	Recurrent or metastatic TN	Phase II, randomized *N* = 56	Primary endpoint: ORR	Status: Active Start: Dec 2020; End: Jan 2025
JAVELIN BRCA/ATM NCT 03565991 (90)	Talazoparib 1 mg day1–28 + avelumab 800 mg every 2 weeks	Locally advanced or metastatic solid tumors with BRCA or ATM defect	Phase II, single-arm study *N* = 202	Primary endpoint: ORR Secondary: TTR, DOR, PFS, OS	Status: active not recruiting Active Start: Jun 2018; End: May 2021
TALAVE NCT03964532 (91)	Talazoparib induction 1 mg daily p.o. D 1-28 for cycle 1, from cycle 2 and subsequently: talazoparib same doses and avelumab i.v. 800 mg every 2 weeks	Advanced breast cancer not amenable of curative intent	Phase I/II, pilot trial N: 24	Primary: safety and tolerability Secondary: ORR	Status: Active, recruiting Start: Apr 2019 End: May 2021
TARA NCT04690855 (92)	Talazoparib + radiotherapy + atezolizumab	Metastatic TN gBRCA 1,2 negative; PD-L1 positive	Phase II; *N* =	Primary endpoint: ORR Secondary: safety, PFS, OS, DoR, TTP	Status: Active, recruiting Start: Apr 2021; End: Apr 2023
SHR-1210 + apatinib and fluzoparib NCT03945604 (93)	SHR-1210 (anti-PD-1 antibody) i.v. in combination with apatinib PO and fluzoparib PO	Recurrent and metastatic triple negative breast cancer	Phase Ib, open-labeled, multi-center, dose-exploring trial N. patients: 52	Primary: DLT (dose-limiting toxicity) Secondary: AEs and SAEs, ORR, DoR, DCR, PFS, 12-months OS rate	Status: active, recruiting Start: Jun 2019 End: Dec 2020

BC, breast cancer; CBR, clinical benefit rate; DDFS, distant disease-free survival; DoR, duration of response; gBRCA, germline BRCA; HRD, homologous recombinant deficiency; HRQoL, health-related quality of life; ORR, overall response rate; OS, overall survival; pCR, pathologic complete response; PFS, progression-free survival; TDT, time to study treatment discontinuation or death; TN, triple negative; TPC, treatment physician’s choice; TFST, time to first subsequent treatment or death; TSST, time to second subsequent treatment or death; TTR, time to response.

**Table 4:** Clinical trial with PARPib plus chemotherapy.

Trial	PARP inhibitor	Setting	Trial characteristics	End points	Study start date (study end)
BROCADE-3 NCT02163694 (94)	Veliparib+ carboplatin d1q21 + paclitaxel weekly *vs*. placebo + carboplatin d1q21 + paclitaxel weekly	HER2 negative germline BRCA mutated breast cancer	Phase III, randomized	Primary endpoint: PFS; Secondary endpoint: OS, CBR, ORR, PFSII *N* = 513	Status: active not recruiting Start: Jun 2014 End: August 2020 (last update)
Veliparib and carboplatin NCT01149083 (95)	Veliparib PO BID on days 1–21 (arm 1) *vs*. carboplatin IV on day 1 and veliparib as in arm 1 (arm 2)	Recurrent stage IIIB, stage IIIC, or stage IV, BRCA-mutated, BC	Phase II, randomized, open label N. patients: 71	Primary: efficacy of single agent veliparib by RR Secondary: PFS, safety, and tolerability of veliparib with or without carboplatin in BRCA mutated, pharmacokinetics, biomarkers analysis	Status: active, Not recruiting Start: Jun 2010 End: Dec 2020
Veliparib and temozolomide NCT01009788 (96)	Veliparib PO twice a day on days 1–7 of each 28 day cycle + temozolomide orally once a day on days 1–5 of a 28-day cycle	Different subtypes of metastatic breast cancer, expanded cohort of BRCA 1/2 mutation carriers	Phase II, single group, open-label N. patients: 64	Primary: ORR, safety, and efficacy in BRCA 1/2 mutation carriers Secondary: safety and tolerability in combination therapy, PFS, CBR	Status: Active, not recruiting Start: Nov 2009 End: Dec 2021
Cisplatin with or without veliparib NCT02595905 (97)	Cisplatin IV on day 1 and placebo PO BID on days 1-14 (arm 1) *vs*. cisplatin IV over 1 h on day 1 and veliparib PO BID on days 1–14	Recurrent or metastatic triple-negative breast cancer, with or without BRCA mutation, with or without brain metastases	Phase II randomized placebo-controlled trial N. patients: 333	Primary: PFS Secondary: OS, response rate, CBR	Status: active, not recruiting Start: Jul 2016 End: Oct 2021

BC, breast cancer; CBR, clinical benefit rate; DoR, duration of response; gBRCA, germline BRCA; HRD, homologous recombinant deficiency; HRQoL, health-related quality of life; ORR, overall response rate; OS, overall survival; PFS, progression-free survival; TN, triple negative; TPC, treatment physician’s choice; TFST, time to first subsequent treatment or death; TSST, time to second subsequent treatment or death.

**Table 5:** Clinical trial with PARPib plus targeted agents.

Trial	PARP inhibitor	Setting	Trial characteristics	End points	Study start date (study end)
VIOLETTE NCT03330847 (98)	Olaparib 300 mg *versus* olaparib 300 mg + ceralasertib *versus* olaparib 300 mg + adavosertib	Metastatic breast cancer-stratified HR-related genes	Phase II randomized; *N* = 273	Primary endpoint: PFS; secondary: ORR; DoR; OS; safety	Status: active not recruiting Start: Jun 2017; End: Sep 2021
SEASTAR NCT03992131 (99)	Rucaparib + sacituzumab govitecan	Advanced solid tumor with deleterious mutation in BRCA1/2, PALB2, RAD51C, RAD51D including TN breast cancer	Phase I–II, *N* = 329	Primary endpoint: safety, ORR; Secondary endpoint: DoR, PFS	Status: Active nor recruiting Start: Jun 2019 End: Mar 2024
NCT03901469 (100)	ZEN 003694 (bromo-domain inhibitor) + talazoparib 1 mg	Pretreated metastatic triple negative breast with no gBRCA1/2 mutation	Phase II, not randomized *N* = 49	Primary endpoint: safety, tolerability, ORR Secondary; pharmacokinetic analysis, TTP, PFS, DoR, QoL	Status: recruiting Start: June 2019 End: Jan 2022
NCT03911973 (101)	Talazoparib 1 mg + getatolisib (PI3K and mTOR inhibitor)	Advanced HER2-negative breast cancer, including TN	Phase I, II *N* = 54	Primary endpoint: safety; ORR Secondary: PFS, DoR, OS, CBR	Status: recruiting Start: Apr 2019; End. May 2022
OPHELIA NCT03931551 (102)	Olaparib 300 mg bid + trastuzumab 4 mg/kg followed by 2 mg/kg or 600 mg subcutaneous q21 days	Metastatic HER2-positive BRCA-mutated BC	Phase II, single arm *N* = 20	Primary endpoint: CBR; Secondary: ORR, PFS, DoR, OS, Safety, HRQoL	Status: recruiting Start: Apr 2019; End Nov 2020
NCT02158507 (103)	Veliparib + lapatinib	Metastatic HER2-positive BRCA-mutated BC	Pilot study; *N* = 23	Primary endpoint: safety Secondary endpoint: ORR, PFS	Status active not recruiting Start: July 2014; End: Dec 2020

BC, breast cancer; CBR, clinical benefit rate; DoR, duration of response; gBRCA, germline BRCA; HRD, homologous recombinant deficiency; HRQoL, health-related quality of life; ORR, overall response rate; OS, overall survival; PFS, progression-free survival; TN, triple negative; TFST, time to first subsequent treatment or death; TTP, time to progression.

### PARP Inhibitors in Combination With Immune Checkpoint Inhibitors

The combination between a PARPi and ICI is based on the evidence of the interaction between the abnormal presence of unrepaired DNA in the cytoplasm of TN tumor cells and the activation of the stimulator of interferon genes pathway which leads to the release of interferons and enhances T-cell infiltration inside the tumor (104). Thus, combining ICIs with a PARPib could be a great strategy to improve the antitumor immunity as well as response to treatment. Promising efficacy and safety findings have been reported in two single-armed phase 2 studies: TOPACIO and MEDIOLA for niraparib combined with pembrolizumab and for olaparib plus durvalumab, respectively (78, 79, 102, 103). The TOPACIO trial enrolled 55 patients of whom 15 were with *BRCA* mutations (103). Overall, an ORR of 21% (47% in patients with *BRCA*-mutated tumors) and a disease control rate (DCR) of 49% (80% in patients with *BRCA* mutated tumors) were reported. For the five patients harboring non-*BRCA* HRR pathway mutations, ORR was 20% (*n* = 1/5) and DCR was 80% (*n* = 4/5). In the overall population, ORR was higher in patients with PD-L1-positive TNBC (32%; *n* = 9/28) than in those with PD-L1-negative tumor (8%; *n* = 1/13). Despite the relatively small sample size (*N* = 47 for efficacy, *N* = 55 for safety), the combination of niraparib and pembrolizumab resulted to be active, regardless of *BRCA* mutation status, in patients with somatic or g*BRCA*-mutated and wild-type BRCA advanced/metastatic TNBC. Comparable results were obtained in the MEDIOLA trial where the combination of olaparib and durvalumab was associated with DCRs of 80 and 50% after 12 and 28 weeks, respectively, and a favorable tolerability in patients with g*BRCA*-mutated metastatic BC (101, 102).

### PARP Inhibitors and Chemotherapy

PARPib are also being evaluated in combination with chemotherapeutic agents (91–94). In the phase 3 BROCADE3 trial (*N* = 509), addition of veliparib to carboplatin and paclitaxel resulted in a significant improvement in median PFS compared with placebo plus carboplatin and paclitaxel (14.5 *vs*. 12.6 months; HR: 0.71, 95% CI: 0.57–0.88; *p* = 0.002) in patients with g*BRCA*-mutated, HER2-negative, locally advanced or metastatic BC (105, 106). The PFS benefit was durable, and no additional toxicities were seen, although there was a high degree of toxicity in both treatment arms (105). However, veliparib appeared to be effective in terms of PFS benefit as monotherapy (HR: 0.49, 95% CI: 0.33–0.73) as well as in combination therapy (HR: 0.81, 95% CI: 0.62–1.06), regardless of the number of treatment cycles. Other ongoing phase II and phase III trials are reported in **Table 4**.

### PARP Inhibitors and Targeted Therapy

Ongoing clinical trials are investigating PARPib in combination with new agents, including DDR molecules (ATR or Wee1 inhibitors). WEE1 is a kinase inhibitor which decreases kinases cyclin-dependent kinase1 (CDK1) expression, subsequently followed by activating replication firing and DSB repair (107). HR is scheduled but weakened by WEE1 inhibitor through phosphorylation of CDK1 in *BRCA*1/2-deficient tumor cells (108, 109). The combination of PARP and WEE1 inhibitors arrests G2 phase and results in chromosomal aberration and replication stress, which is proven to have an antitumor activity in numerous preclinical models (110). VIOLETTE is a global, multicenter, open-label, phase II study randomizing 1:1:1 450 patients with advanced TNBC to olaparib alone or in combination with AZD1775 (a WEE1 checkpoint inhibitor) or AZD6738 (an ataxia telangiectasia and Rad3-related protein inhibitor). Patients will be stratified in *BRCA*-mutated, non-*BRCA* HRR-mutated, and non-HRR mutated. The primary endpoint is PFS (95).

A two-part, open-label, non-randomized, phase 2, ongoing trial is testing the combination of ZEN003694 (a bromodomain inhibitor) with talazoparib in patients with TNBC without *BRCA* 1/2 germline mutations. The part 1 of this trial is a dose escalation study, with primary outcome incidence of treatment-related adverse events and treatment-related serious adverse events. The part 2 is a Simon 2-stage design, with primary outcome ORR (97).

The other group of agents that are interesting are the AKT inhibitors: previous research has shown that PI3K inhibitors (PI3Kib) lower nucleotide pools required for DNA synthesis and S-phase progression. Additionally, inhibition of PI3K/mTOR could inhibit PI3K interaction with the homologous recombination complex, increasing the dependency on PARP enzymes for DNA repair (111). Based on this data, the combination of PI3Kib and PARPib could potentially lead to a new, chemotherapy-free treatment option for *BRCA* wild-type TNBC as well as to improve the modest PFS/OS seen with the PARPib as single agents in *BRCA*1/2 mutant advanced setting. At the ASCO 2020, two randomized phase 2 studies, LOTUS and PAKT, reported the role of AKT inhibitors in combination with taxanes. Both trials demonstrated some improvement in PFS, with hints toward improvement in OS, in advanced TNBC. The results also showed some suggestions that PTEN loss or a PI3K-altered pathway could be a biomarker to predict who is going to benefit the most AKT inhibitors. Thus, a dual mTOR/PI3K inhibitor (gedatolisib) for metastatic or recurrent/unresectable TNBC could be a promising strategy in combination with talazoparib (98). Finally, the combination of olaparib plus trastuzumab for HER2-positive BC (OPHELIA trial) and a phase I trial with veliparib plus lapatinib are also under evaluation (99, 100). A complete list of clinical trials evaluating the combination of PARPib with other targeted therapies is summarized in **Table 5**.

### PARP Inhibitors in Triple Negative Breast Cancer Beyond *BRCA* Mutations

Along with *BRCA*1 and *BRCA*2, multiple HRR genes, including *ATM*, *BARD1*, *BRIP1*, *CHEK2* (encodes CHK2), *MRE11A*, *PALB2*, *RAD50*, *RAD51C*, and *RAD51D*, are also implicated in hereditary cancer risk and are recently considered new potential biomarkers in patients with non-g*BRCA* HRR gene mutations (112).

Clinical studies that showed positive findings for PARPib in settings other than g*BRCA*-mutated BC include single-arm phase 2 studies of olaparib (113), rucaparib (114), and talazoparib (75) monotherapy (**Table 6**).

**Table 6:** Clinical trial with PARP inhibitors in HRD-defective triple negative breast cancer.

Trial	PARP inhibitor	Setting	Trial characteristics	End points	Study start date (study end)
TBCRC048 (104)	Olaparib 300 mg bid/day	Metastatic BC with germline or somatic mutation in HRD	Phase II, single arm *N* = 114	Primary endpoint: safety, ORR; Secondary endpoint: CBR, PFS, safety	Status: Recruiting Start: Nov 2017 End: Apr 2021
RUBY trial NCT02505048 (105)	Rucaparib 600 mg bid/day	HER2-negative metastatic breast cancer with BRCAness genomic signature	Phase II single arm; *N* = 41	Primary endpoint: CBR; Secondary: ORR; OS, PFS; safety	Status: completed Start: Jun 2015; End: Jun 2021 (last update
NCT 02401347 (106)	Talazoparib 1 mg	HER2-negative metastatic BC in BRCA1/2 WT, HRD	Phase II, single arm *N* = 40	Primary endpoint: ORR Secondary: CBR, PFS, safety	Status: recruiting. Start: Mar 2015; Last update: Aug 2020

BC, breast cancer; CBR, clinical benefit rate; HRD, homologous recombinant deficiency; ORR, overall response rate; OS, overall survival; PFS, progression-free survival; WT, wild type.

In the olaparib expanded study, in 54 patients with metastatic BC and germline mutations in various non-BRCA DDR genes (cohort 1) or somatic mutations in DDR genes including BRCA (cohort 2), ORR was 33 and 31%, respectively (115). Overall, antitumor activity was reported in patients with somatic *BRCA* or g*PALB2* mutations, but not in those with *ATM* or *CHEK2* mutations. At the ASCO 2020 symposium, a study investigating the role of olaparib in women with HER2-negative breast cancer and a germline alteration in DDR pathway, such as *PALB2*, *CHEK2*, and *ATM*, or a somatic tumor mutation without a germline BRCA1/2 mutation was presented (115). Of the two cohorts, the first included patients with germline mutations other than BRCA. Olaparib demonstrated a high response rate, and the trial met its primary endpoint. Specifically, patients with a germline *PALB2* mutation had 80% ORR, whereas in the somatic mutation cohort, patients with a somatic *BRCA*1/2 mutation reported 50% ORR.

In the RUBY trial, rucaparib monotherapy was investigated in 41 patients with HRD, including four patients harboring somatic BRCA mutations. Five patients (13.5%) demonstrated clinical benefit, comprising three patients with high loss of heterozygosity, one with a somatic BRCA1 mutation, and another patient with a somatic BRCA2 mutation (116).

In the phase 2 study of single-agent talazoparib, patients with BRCA wild-type, HER2-negative, advanced BC and non-BRCA HRR pathway mutations were enrolled. Based on 12 evaluable patients, the ORR and the clinical benefit rate were 25 and 50% after 6 months of treatment, respectively (117). In detail, two-thirds of the responders had gPALB2 mutations; the others had gCHEK2, gFANCA, and somatic PTEN mutations.

According to the above-mentioned reported data, PARPib demonstrated to have a role beyond BRCA2 germline mutation carriers, although the responses seem to be gene specific: the ATM and CHEK2 cohorts seemed not to respond, but the sample size was small.

## Discussion

Olaparib and talazoparib are now approved for triple negative metastatic breast cancer patients harboring gBRCA 1 or 2 mutations. Both registered trials (OlympiAD and EMBRACA) showed a consistent PFS benefit when compared to chemotherapy (7.0 *versus* 4.2 months for olaparib, HR: 0.58; 95% CI: 0.43 to 0.80; *p* < 0.001; 8.6 *versus* 5.6 months for talazoparib, HR: 0.54; 95% CI, 0.41–0.71; *p* = 0.001) (36, 37). However, the PFS benefit did not translate in a significant OS benefit for either of the two trials (66, 71). In fact, findings from a final prespecified analysis showed no OS difference in the general population (19.3 *versus* 17.1 months; HR: 0.90; 95% CI: 0.66–1.23; *P* = 0.513 for olaparib; 19.3 *versus* 19.5 months for talazoparib, HR: 0.848; 95% CI, 0.670–1.073; *p* = 0.17) and in the TNBC subgroup (18.8 *versus* 17.2 months; HR: 1.13; 95% CI: 0.79–1.64; *P* = NS for olaparib; data not available for talazoparib). A possible reason that could explain the OS lack of benefit is that the sample was not powered to detect OS differences between the two arms, as it was in the OlympiAD trial, or crossover design: in the EMBRACA trial, approximately 35% of patients treated in the control arm received a PARPib in subsequent lines of therapy *versus* 8% of patients enrolled in the OlympiAD trial. Interestingly, in the OlympiAD trial, a 7.9-month OS benefit was observed in patients who had not received prior chemotherapy for metastatic breast cancer, confirming previously published data where olaparib seemed to be more active in less pretreated patients (38). However, the sample size was small. Therefore, a confounding process cannot be excluded. Furthermore, perspective data are needed to confirm this finding. The safety profile for both talazoparib and olaparib was manageable: drug discontinuation was low (<5% for olaparib and 5.9% for talazoparib), showing that supportive therapies and dose interruptions/reductions were sufficiently effective to manage tolerability (66, 118). Most grade 3 and 4 adverse events were hematological: 40% of patients in the talazoparib arm and 16% of patients in the olaparib arm experienced grade 3 anemia. Fortunately, no new side effects were recorded with extended follow-up, and the safety profile was consistent with the primary analysis, indicating the absence of cumulative toxicity with prolonged exposition to the molecules.

Recent results have demonstrated that PARP inhibitors could play an emerging role in the maintenance treatment of advanced ovarian cancer with long-term efficacy and improved PFS in patients with newly diagnosed disease experimenting CR or PR to platinum-based chemotherapy (119–121). According to these results and in the light of the emerging role of PARP inhibitors in the treatment of triple negative breast cancer, there is a solid scientific rationale for the use of these molecules as maintenance therapy even in patients with TNBC.

One of the most consequential risks associated with PARPib is the development of therapy-related myeloid neoplasms (t-MNs). Most of the data available about t-MNs come from ovarian cancer where incidence is estimated in 1–3% of patients (122, 123). The spectrum of t-MNs comprehends myelodysplastic syndrome (MDS) and acute myeloid leukemia (AML). Both are characterized by a complex karyotype and poor prognosis (124), but the mechanisms responsible for t-MNs onset have not yet been clarified. In fact, a recent metanalysis did not confirm the association between t-MNs and previously identified clinical risk factors such as g*BRCA* variants, recurrent disease, and exposure to specific antineoplastic agents (125). According to a recently published systematic review, which evaluated the safety profile of 31 RCTs comparing PARPib therapy *versus* control treatments in different settings and tumor types, PARPib therapy was associated with an increased risk of t-MNs, but all the cases of MDS or AML were reported in RCTs in ovarian cancer (126). This exclusivity for ovarian cancer might be explained by the difference in median follow-up, with ovarian cancer RCTs having the longest duration when compared to the other trials included in the analysis. Therefore, at this point, patients with metastatic TNBC treated with PARPib do not seem to be at a higher risk for t-MN development, but a longer follow-up is needed to confirm those findings.

Despite the established role of PARPib in the therapeutical armamentarium of TNBC treatment, almost all the patients will become eventually resistant to the therapy, thus the need to improve therapeutical opportunities for this class of patients. In recent years, precision medicine is rapidly evolving thanks to next-generation sequencing (NGS) advances. Genomically driven molecular interrogation revealed that TNBC is a complex and heterogeneous disease. Unfortunately, there is still lack of clinical data supporting a major benefit of PARPib therapy in specific TN molecular subtypes (*e*.*g*., immunomodulatory, basal-like, *etc.*), but recent evidences showed that approximately 20% of patients with basal-like tumors harbored genetic or somatic BRCA1/2 mutations which may confer sensitivity to PARPib or platinum compounds (127, 128).

To overcame drug resistance and take advantage from our better understanding of TN tumors, an optimized and effective strategy probably requires a treatment combination rather than monotherapies. In that sense, several ongoing trials are combining PARPib with other agents (**Tables 3**–**5**). At this time, major clinical evidences derive from the combination of PARPib with ICIs: two phase 2, single-arm studies (TOPACIO and MEDIOLA) showed comparable promising results in terms of ORR, safety, and tolerability with the combination of niraparib plus pembrolizumab (101) and durvalumab plus olaparib (102), respectively. Alongside this, several phase I and II trials are evaluating PARPib with other targeted agents according to the growing stratification and knowledge of TNBC chromosomal aberrations. Hopefully, in the near future, the role of PARPib for the treatment of TNBC will gradually evolve towards a more personalized approach with promising expectations.

## Conclusion

PARPib now represent a standard of care for the treatment of patients with triple negative breast cancer and gBRCA mutations. The oral formulation and the improvement in QoL are responsible for the increasing adherence and awareness of the patients. The safety profile is manageable, but patients must be checked routinely. Future directions comprehend the association of PARPib with other agents such as immunotherapy and other targeted therapies and the inclusion of patients with somatic BRCA mutations or patients carrying mutations beyond BRCA1 and BRCA2 but always involved in HRR pathway.

## Author Contributions

GB and MR wrote the manuscript. MV selected bibliographic contributes. PV corrected the manuscript. ST checked the introduction, the conclusions, and the tables. FT devised, designed, and supervised the paper. All authors contributed to the article and approved the submitted version.

## Conflict of Interest

The authors declare that the research was conducted in the absence of any commercial or financial relationships that could be construed as a potential conflict of interest.

## Publisher’s Note

All claims expressed in this article are solely those of the authors and do not necessarily represent those of their affiliated organizations, or those of the publisher, the editors and the reviewers. Any product that may be evaluated in this article, or claim that may be made by its manufacturer, is not guaranteed or endorsed by the publisher.

## Abbreviations

AML, acute myeloid leukemia; CSC, cancer stem cells; DSB, double-strand breaks; HR, homologous recombination; HR+, hormone receptors—positive; HRR, homologous recombination repair; HRQoL, health-related quality of life; ICIs, immune checkpoint inhibitors; MDS, myelodysplastic syndrome; NGS, next-generation sequencing; NHEJ, non-homologous end-joining; ORR, overall response rate; OS, overall survival; PARPib, PARP inhibitors; PI3Kib, PI3K inhibitors; PFS, progression-free survival; RCTs, randomized clinical trials; SSB, single-strand breaks; t-MNs, therapy-related myeloid neoplasm; TN, triple negative; TNBC, triple negative breast cancer; TPC, treatment physician’s choice.
